# Genome-wide analysis and expression profiling suggest diverse roles of *GH3* genes during development and abiotic stress responses in legumes

**DOI:** 10.3389/fpls.2014.00789

**Published:** 2015-01-14

**Authors:** Vikash K. Singh, Mukesh Jain, Rohini Garg

**Affiliations:** Functional and Applied Genomics Laboratory, National Institute of Plant Genome ResearchNew Delhi, India

**Keywords:** gene family, *GH3* genes, legumes, RNA-seq, homology modeling, substrate specificities

## Abstract

Growth hormone auxin regulates various cellular processes by altering the expression of diverse genes in plants. Among various auxin-responsive genes, *GH3* genes maintain endogenous auxin homeostasis by conjugating excess of auxin with amino acids. *GH3* genes have been characterized in many plant species, but not in legumes. In the present work, we identified members of GH3 gene family and analyzed their chromosomal distribution, gene structure, gene duplication and phylogenetic analysis in different legumes, including chickpea, soybean, *Medicago*, and *Lotus*. A comprehensive expression analysis in different vegetative and reproductive tissues/stages revealed that many of *GH3* genes were expressed in a tissue-specific manner. Notably, chickpea *CaGH3-3*, soybean *GmGH3-8* and *-25*, and *Lotus LjGH3-4*, *-5*, *-9* and *-18* genes were up-regulated in root, indicating their putative role in root development. In addition, chickpea *CaGH3-1* and *-7*, and *Medicago MtGH3-7*, *-8*, and *-9* were found to be highly induced under drought and/or salt stresses, suggesting their role in abiotic stress responses. We also observed the examples of differential expression pattern of duplicated *GH3* genes in soybean, indicating their functional diversification. Furthermore, analyses of three-dimensional structures, active site residues and ligand preferences provided molecular insights into function of *GH3* genes in legumes. The analysis presented here would help in investigation of precise function of *GH3* genes in legumes during development and stress conditions.

## INTRODUCTION

Auxin is an important phytohormone which regulates various aspects of plant growth and development. Most of these processes are regulated by auxin-responsive genes, namely auxin/indole-3-acetic acid (Aux/IAA), auxin-response factor (ARF), small auxin-up RNAs (SAUR) and Gretchen Hagen3 (GH3; Hagen and Guilfoyle, 2002). Auxin-responsiveness to these genes is conferred by auxin-responsive elements (AuxREs, TGTCTC) present in their promoters (Hagen et al., 1991; Li et al., 1994; Ulmasov et al., 1995; Hagen and Guilfoyle, 2002). To understand molecular mechanism of auxin action, several auxin-responsive genes have been isolated and characterized from many plant species, such as pea, soybean, tobacco, and cucumber (Hagen and Guilfoyle, 2002).

Gretchen Hagen3 family of proteins maintain auxin level by catalyzing conjugation of amino acids with indole-3-acetic acid, salicylic acid (SA), and jasmonic acid (JA; Staswick et al., 2002, 2005). The first *GH3* gene was identified by Hagen et al. (1984) as an early auxin-responsive gene in soybean. Since then, a large number of *GH3* homologs have been identified in numerous plant species ranging from mosses to angiosperms (Jain et al., 2006; Terol et al., 2006; Ludwig-Müller et al., 2008; Kumar et al., 2012; Yuan et al., 2013). The studies on GH3 proteins have revealed their regulatory function in plant growth, organ development, light signaling, abiotic stress tolerance, and plant defense responses (Woodward and Bartel, 2005; Park et al., 2007; Jain and Khurana, 2009; Ludwig-Muller, 2011; Du et al., 2012; Kumar et al., 2012; Yuan et al., 2013). In *Arabidopsis*, GH3 gene family has been classified into three groups (I–III) based on sequence similarity and substrate specificities (Staswick et al., 2002). Group I GH3 proteins of *Arabidopsis* are JA-amido synthetases (Staswick et al., 2002, 2005). AtGH3-11, a group I GH3 protein, was characterized based on analysis of *jar1* mutant, which was insensitive to JA and was required for the formation of bioactive jasmonate JA-isoleucine (Staswick et al., 2002). A different allele of this gene (*FIN219*) was identified as a phytochrome A signaling component, having crucial role in photomorphogenesis (Hsieh et al., 2000). Group II GH3 proteins of *Arabidopsis* are involved in conjugation of IAA to various amino acids (Staswick et al., 2002, 2005). *AtGH3-2* gain-of-function mutant, *Ydk1-D*, was shown to be responsible for short primary root, reduced lateral root number, and apical dominance (Takase et al., 2004). In another report *AtGH3-6* mutant, *dfl1*, was shown to regulate shoot elongation and lateral root formation negatively, but positively regulate the light responses to hypocotyl length (Nakazawa et al., 2001). Some Group II GH3 proteins of rice (TLD1/OsGH3-13, OsGH3-2, and OsGH3-8) have also been characterized that conjugate IAA with aspartate or alanine (Chen et al., 2009, 2010; Zhang et al., 2009). A gain-of-function mutant of rice *OsGH3-13* gene, *tld1-D*, resulted in increased tillers, enlarged leaf angles, dwarfism and improved drought tolerance (Zhang et al., 2009). AtGH3-12/PBS3 is the only characterized member of group III, which catalyzes the conjugation of glutamic acid (Glu) to 4-aminobenzoate and 4-hydroxybenzoate and is involved in SA signaling (Jagadeeswaran et al., 2007; Nobuta et al., 2007; Okrent et al., 2009). Recently, the crystal structure and mechanism of catalytic action of AtGH3-12 and JAR1/AtGH3-11 (Westfall et al., 2012) in *Arabidopsis*, and VvGH3-1 in grapevine (Peat et al., 2012) have also been reported.

Legumes are nutritionally important crop plants, which serve as a rich source of proteins and fibers. Although the first auxin-responsive gene was identified from soybean (Hagen et al., 1984), genome-wide analysis of *GH3* genes in legumes is still lacking. This may be attributed to scarcity of genomic resources for legumes until recently. However, in recent years several genomic resources have been generated for various legumes. The genome and transcriptome sequences of desi and kabuli chickpea (*Cicer arietinum*), soybean (*Glycine max*), *Medicago* (*Medicago truncatula*), and *Lotus* (*Lotus japonicus*) have been published (Sato et al., 2008; Schmutz et al., 2010; Garg et al., 2011; Young et al., 2011; Jain et al., 2013; Varshney et al., 2013). The availability of genome annotation provides an opportunity for characterization of GH3 gene family in legumes, which can help in better understanding of their function in various cellular processes. The availability of crystal structures of GH3 proteins (Peat et al., 2012; Westfall et al., 2012) provides a resource to identify substrate specificity determining motifs/residues in the GH3 proteins, which can help in understanding auxin-mediated regulation of cellular processes in legumes.

Here, we performed genome-wide identification and analysis of GH3 gene family in four legume species, including chickpea, soybean, *Medicago,* and *Lotus*. We report their genomic organization, chromosomal distribution, sequence homology, and phylogenetic relationship in/among different legumes. Comprehensive gene expression analyses in various tissues/stages and abiotic stress conditions have also been performed to gain insight into their putative function. Putative promoter sequences of the *GH3* genes were also analyzed for identification of *cis*-regulatory elements, which may be involved in various development processes and stress responses. In addition, their ligand preferences were predicted based on the protein structure and sequence analysis. These data provide a framework for further in-depth functional analyses of *GH3* genes in legumes.

## MATERIAL AND METHODS

### IDENTIFICATION OF *GH3* GENES

Chickpea genome annotation was downloaded from Chickpea Genome Analysis Project (CGAP v1.0; Jain et al., 2013), soybean and *Medicago* genome annotations were downloaded from Phytozome (v9.0^1^), and *Lotus* genome annotation was taken from miyakazusa.jp database (v2.5^2^). A total of 19 protein sequences of GH3 family members of *Arabidopsis* and 13 protein sequences of rice GH3 family members were downloaded from TAIR^3^ and RGAP database^4^, respectively. The rice and *Arabidopsis* GH3 proteins were searched in chickpea, soybean, *Medicago* and *Lotus* proteomes individually, using BLASTP with an *e*-value cutoff of 1e-05. Further, the HMM profile of GH3 domain was downloaded from pfam database^5^ and HMMER was used to search proteomes of chickpea, soybean, *Medicago,* and *Lotus* for GH3 domain. All the tentative gene lists obtained from these two searches were combined to make a non-redundant gene list for each legume, and their protein sequences were searched in pfam database to confirm the presence of conserved GH3 domain. Using the similar strategies, we investigated the chickpea transcriptome sequence (Garg et al., 2010) as well for identification of any additional GH3 gene family member that may not be represented in chickpea genome annotation.

### SEQUENCE ANALYSIS AND PHYLOGENETIC TREE CONSTRUCTION

Multiple sequence alignment of all the GH3 protein sequences of chickpea, soybean, *Medicago* and *Lotus* with *Arabidopsis* GH3 protein sequences was carried out using MAFFT and phylogenetic tree was constructed by UPGMA method using CLC Genomics Workbench (v4.7.2). Bootstrap analysis was performed by taking 1,000 replicates and the generated tree was viewed using FigTree (v1.3.1).

### GENE DUPLICATION ANALYSIS

Synteny analysis was performed using Plant Genome Duplication Database^6^. Syntenic blocks were evaluated using Circos tool. Information about the chromosome locations was obtained from Phytozome database. Genes were regarded as segmentally duplicated if they found to be coparalogs located on duplicated blocks, as proposed by Wei et al. (2007). Tandem duplication was characterized as multiple genes of one family located within the same or neighboring intergenic region (Du et al., 2013a).

### PROMOTER SEQUENCE ANALYSIS

Genomic co-ordinates of coding sequences were determined using GFF files obtained from chickpea and soybean genome annotation. The regions of 2,000 bp upstream from start codon were extracted from genomic DNA sequences. *Cis*-regulatory elements on both strands of promoter sequences were scanned using PLACE web server^7^ .

### HOMOLOGY MODELING

The 3-D protein structures of AtGH3-11 (Protein Data Bank code 4EPL; Westfall et al., 2012) and Vv-GH3-1 (Protein Data Bank code 4B2G; Peat et al., 2012) were downloaded from Protein Data Bank^8^. Phyre2 (Protein Homology/AnalogY Recognition Engine^9^) was used for predicting the protein structure by homology modeling under ‘intensive’ mode (Kelley and Sternberg, 2009). The protein structures modeled with >90% confidence were selected. The core of predicted protein structure or allowed area in the plot showing the preferred region for psi/phi angles pair for residues was determined through Ramachandran plot using RAMPAGE server^10^ and models were viewed using Chimera (V1.9). Only those structures representing >95% of residues in favored region were considered for further analysis. For substrate binding site prediction, templates and model were superimposed using MatchMaker of Chimera (V1.9) and ligands were transferred on model from templates.

### PLANT MATERIAL AND STRESS TREATMENTS

Chickpea (*C. arietinum* L. genotype ICC4958) seeds were grown in culture room and field for collection of various tissue samples. Mature leaf, young leaf, young pod, flower buds (FB), flower bud opened (FBO), unopened flowers (UOF), and mature flower (MF) were harvested from field grown plants. Root and shoot tissues were collected from 15-day-old chickpea seedlings grown in autoclaved mixture (1:1) of agropeat and vermiculite in plastic pots in the culture room maintained at 22 ± 1°C with a photoperiod of 14 h, as described (Garg et al., 2010). Germinating seedlings (GS) were collected after 5 days of seed germination on wet Whatman paper sheet in Petri dishes as described (Singh et al., 2013). Two stages of flower bud development (FB 4 mm and FBO 8–10 mm) were collected on the basis of size and morphological differences (Singh et al., 2013). Two stages of flower development, including young flower with closed petals (UOF) and MF with opened petals were also collected. For stress treatments, 10-day-old chickpea seedlings were kept in water for control, 150 mM solution of NaCl for salt stress, at 4°C for cold stress and between folds of tissue paper for desiccation stress. Root and shoot tissues were harvested separately after 5 h of treatments as described (Garg et al., 2010). All samples were quickly frozen into liquid nitrogen after harvesting and stored at -80°C till RNA isolation.

### RNA ISOLATION AND QUANTITATIVE RT-PCR ANALYSIS

Total RNA was extracted using TRI reagent (Sigma Life Science, St. Louis, MO, USA) following the manufacturer’s instructions. RNA quality and quantity was determined using Nanodrop 1000 spectrophotometer (Thermo Fisher Scientific, Wilmington, DE, USA). RNA samples with 260/280 ratio between 1.8 and 2.1 and 260/230 ratio between 2.0 and 2.5 were used for cDNA synthesis. Primers were designed for all genes using Primer Express (v3.0) software (Applied Biosystems, Foster City, CA, USA). Specificity of each pair of primers was determined via BLAST search. All the primer sequences used have been listed in Supplemental Table S1. For each tissue, at least two independent biological replicates and three technical replicates of each biological replicate were taken for the analysis. Real time PCR analysis was performed using the 7500 Detection System (Applied Biosystems) as described (Garg et al., 2010). The expression of *elongation factor-1 alpha* gene was used as internal control for normalization of sample input variance (Garg et al., 2010).

### RNA-seq AND MICROARRAY DATA ANALYSIS

The expression patterns of chickpea and soybean *GH3* genes were analyzed using RNA-seq data from various tissue/stages of development. For chickpea, we mapped our RNA-seq data (Singh et al., 2013) on the genome using TopHat (v2.0.6), assembled with Cuﬄinks (v2.1.1), and merged with Cuffmerge to estimate read count in FPKM. For soybean, normalized gene expression data (RPKM) was downloaded from SoySeq^11^. *Medicago* and *Lotus GH3* gene expression data were downloaded from MtGEA^12^ and LjGEA^13^, respectively. Probsets corresponding to *MtGH3* and *LjGH3* genes were identified using BLASTN search with best hits.

## RESULTS AND DISCUSSION

### GH3 GENE FAMILY IN LEGUMES

The availability of genome sequences provides an opportunity to identify and analyze GH3 gene family in legumes. We investigated members of GH3 gene family in four legumes, including chickpea, soybean, *Medicago,* and *Lotus*, using two strategies, BLASTP and HMM profile search. For chickpea, we selected genome sequence of desi genotype (ICC4958), because of the availability of comprehensive expression (RNA-seq) data from various tissues/developmental stages (Jain et al., 2013) and abiotic stress conditions (Garg et al., 2014), which can provide better insights into the functions of *GH3* genes (as described in latter sections). The GH3 gene family members identified via these two searches were combined and a unique gene list was obtained for each legume species. In total, 11, 28, 10, and 18 *GH3* gene members were identified in chickpea, soybean, *Medicago* and *Lotus*, respectively, after analyzing their protein sequences in pfam database for the presence of conserved GH3 domain. To identify additional members of GH3 gene family in chickpea, which may not be represented in the genome annotation, the published chickpea transcriptome (Garg et al., 2011) was also analyzed using similar strategies. This resulted in the identification of one additional GH3 gene family member for a total of 12 in chickpea. A list of *GH3* genes and their identifiers in different legumes along with their genomic co-ordinates is given in Supplemental Table S2.

The number of GH3 proteins identified in chickpea, *Medicago* and *Lotus* were comparable to *Arabidopsis* (10; excluding group III members, which are exclusively present in *Arabidopsis*), rice (13), tomato (15), and sorghum (16; Jain et al., 2006; Wang et al., 2010; Kumar et al., 2012). Whereas, the number of GmGH3 proteins are found to be approximately double as compared to other legume plants. The soybean genome has undergone two rounds of whole genome duplication, including an ancient duplication prior to the divergence of papilionoids (58–60 Mya) and a soybean-specific duplication that is estimated to have occurred ∼13 Mya (Schmutz et al., 2010), which might have resulted into duplication of members of this gene family.

### GENOMIC ORGANIZATION AND CHROMOSOMAL DISTRIBUTION

**FIGURE 1:**
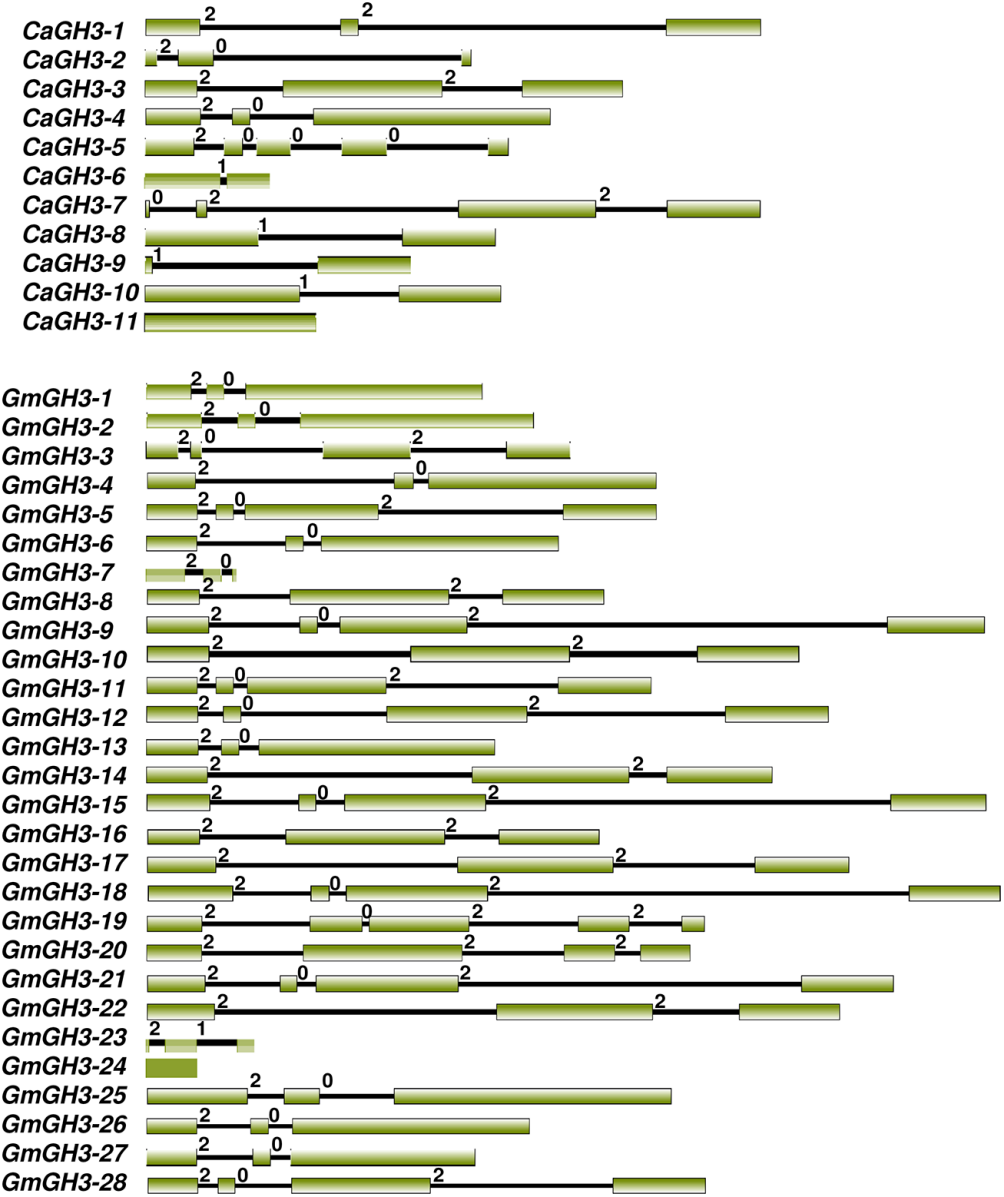
**Exon-intron organization of chickpea and soybean *GH3* genes.** Boxes and lines represent exons and introns, respectively. The numbers 0, 1, and 2 represent phase 0, 1, and 2 introns, respectively.

All GH3 proteins identified in legumes showed the presence of characteristic GH3 domain, and sequence conservation in the core region in multiple sequence alignment of proteins. The gene structure (exon–intron organization) analysis of *CaGH3* and *GmGH3* genes revealed that number of introns varied from one to four except for *CaGH3-11* and *GmGH3-24*, which do not have any intron (**Figure 1**). Most of the *CaGH3* and *GmGH3* had similar intron-phasing distribution (**Figure 1**) and followed the pattern reported earlier for rice *GH3* genes (Jain et al., 2006). Next, we analyzed the distribution of *GH3* genes on the chromosomes in different legumes. Only two *CaGH3* genes could be located on the linkage groups, whereas others were located on scaffolds (Supplemental Table S2). This may be due to availability of incomplete draft genome sequence and unanchored scaffold as of now (Jain et al., 2013). For soybean, all the 28 *GmGH3* genes were distributed on 14 of 20 chromosomes, with six *GH3* genes located on chromosome 12, four and three being present on chromosome 6 and 13, respectively, two each on chromosome 2, 3, 15, and 17, and one each on chromosome 1, 5, 7, 10, 11, 16, and 19 (Supplemental Table S2). In soybean, many *GH3* genes were clustered, such as adjacent genes on chromosome 6 (*GmGH3-7*, *-8*, *-9*, and *-10*), chromosome 12 (*GmGH3-14* and *-15,* and *GmGH3-17*, *-18* and *-19*), chromosome 13 (*GmGH3-20*, *-21*, and *-22*), and chromosome 17 (*GmGH3-26* and *-27*). The amino acid sequences of these genes [GmGH3-7 to -10 (7–8% identity), GmGH3-14, and -15 (32% identity), GmGH3-17, -18, -19 (28–57% identity), GmGH3-20 to -22 (30–68%), and GmGH3-26 and -27 (70% identity)] showed very low (7%) to high (70%) similarity (Supplemental Table S3B), indicating that these *GmGH3* genes probably resulted from tandem duplication and some of them diverged during course of evolution. In *Medicago*, 7 of 10 *GH3* genes were distributed on 5 of 8 chromosomes and three *MtGH3* genes were located on scaffolds (Supplemental Table S2). Chromosome 5 and 8 of *Medicago* harbored two *MtGH3* genes each and one each resided on chromosome 2, 3, and 7. In *Lotus*, out of 18 *LjGH3* genes, only eight were located on 4 of 6 chromosomes; three located on chromosome 3, two each on chromosomes 2 and 4, and one on chromosome 1 (Supplemental Table S2). Altogether, it appears that tandem gene duplication resulted in the amplification of GH3 gene family members in legumes and low homology between them suggested their divergence during course of evolution.

### SEQUENCE ANALYSIS AND PHYLOGENETIC RELATIONSHIP

**FIGURE 2:**
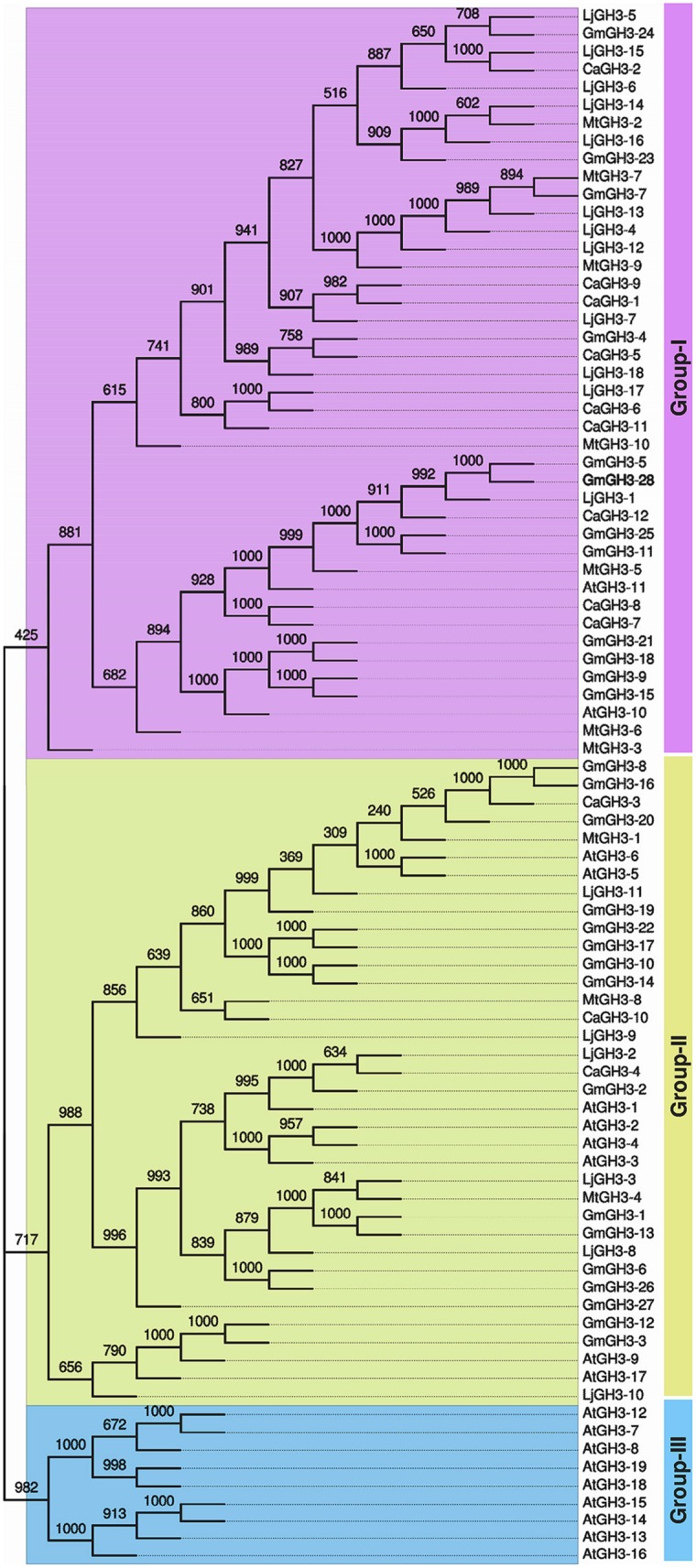
**Phylogenetic relationship among chickpea, soybean, *Medicago*, *Lotus,* and *Arabidopsis* GH3 proteins.** Multiple sequence alignment of all GH3 proteins from chickpea (CaGH3), soybean (GmGH3), *Medicago* (MtGH3), *Lotus* (LjGH3) and *Arabidopsis* (AtGH3) was performed and tree was generated by UPGMA method. FigTree was used for visualization of the tree. The value at the nodes represents bootstrap values from 1000 replicates. Different groups of GH3 proteins are labeled.

Pairwise analysis of the full-length protein sequences of chickpea and soybean GH3 proteins showed very high homology, 76.9% between paralogous pair, CaGH3-7 and CaGH3-8 (**Figure 2**; Supplemental Table S3A), and 97.4% between paralogous pair, GmGH3-8 and GmGH3-16 (**Figure 2**; Supplemental Table S3B). Such high homologies suggest that they may perform similar functions (Jain et al., 2006).

In *Arabidopsis*, GH3 proteins have been classified into three groups on the basis of sequence similarity and specificity to adenylate plant hormones (Staswick et al., 2002). We also analyzed the phylogenetic relationship among GH3 proteins identified in legumes and classified them into different groups. Phylogenetic analysis of legume GH3 proteins showed clustering into only two groups, I and II. Group III GH3 proteins were found absent in all the legumes (**Figure 2**). This observation is consistent to previous reports (Jain et al., 2006; Kumar et al., 2012; Yuan et al., 2013) and suggested that group III GH3 proteins might have been lost in legumes during the course of evolution. The group I consisted of nine members of CaGH3 proteins, 12 GmGH3 proteins, seven MtGH3 proteins, and 12 LjGH3 proteins (**Figure 2**). Group II included three CaGH3 proteins, 16 GmGH3 proteins, three MtGH3 proteins, and six LjGH3 proteins (**Figure 2**).

Phylogenetic tree comprising CaGH3, GmGH3, MtGH3, LjGH3, and AtGH3 proteins showed a total of 26 sister pairs. Group I comprised of 12 sister pairs, four of GmGH3-GmGH3 proteins, two each of CaGH3-CaGH3 and CaGH3-LjGH3 proteins, and one each of CaGH3-GmGH3, GmGH3-MtGH3, GmGH3-LjGH3, MtGH3-LjGH3 proteins. Group II consisted of eleven sister pairs, including six GmGH3-GmGH3 proteins, two each of AtGH3-AtGH3 proteins, and one each of CaGH3-MtGH3, CaGH3-LjGH3, and MtGH3-LjGH3 proteins. In Group III, three sister pairs of only *Arabidopsis* GH3 proteins were present. To gain further insight into structural diversity of *GH3* genes, we compared exon/intron organization of individual *GH3* gene in chickpea and soybean. Most of the sister pairs shared similar exon/intron structures, intron numbers and intron phasing (**Figure 2**; Supplemental Tables S3A,B). However, all closely located *GmGH3* genes, such as *GmGH3-7, -8, -9, -10* on chromosome 6, *GmGH3-17, -18*, and *-19* on chromosome 12, *GmGH3-20, -21*, and *-22* on chromosome 13, and *GmGH3-26* and *-27* on chromosome 17, were not paired together (**Figure 2**). This suggested that these genes might have diverged substantially during evolution. Most of the AtGH3/GmGH3 proteins showed 1:4 orthologous relationship, such as AtGH3-10/GmGH3-9, -15, -21, and -18 (**Figure 2**). Presence of such orthologous relationship between AtGH3/GmGH3 pairs is also in agreement with the fact that soybean whole genome duplication happened twice in the past (Schmutz et al., 2010). Some *Arabidopsis* and legume GH3 protein pairs (AtGH3-5 and -6/CaGH3-3 and -10, AtGH3-9, and -17/GmGH3-3, and -12, AtGH3-11/MtGH3-5, LjGH3-1) exhibited n:n orthologous relationship, which suggest that members of this family have diversified both in *Arabidopsis* and legumes independently (Wang et al., 2007; Wu et al., 2011; Liu and Hu, 2013; Yuan et al., 2013).

### DIFFERENTIAL EXPRESSION PATTERNS OF *GH3* GENES DURING DEVELOPMENT

**FIGURE 3:**
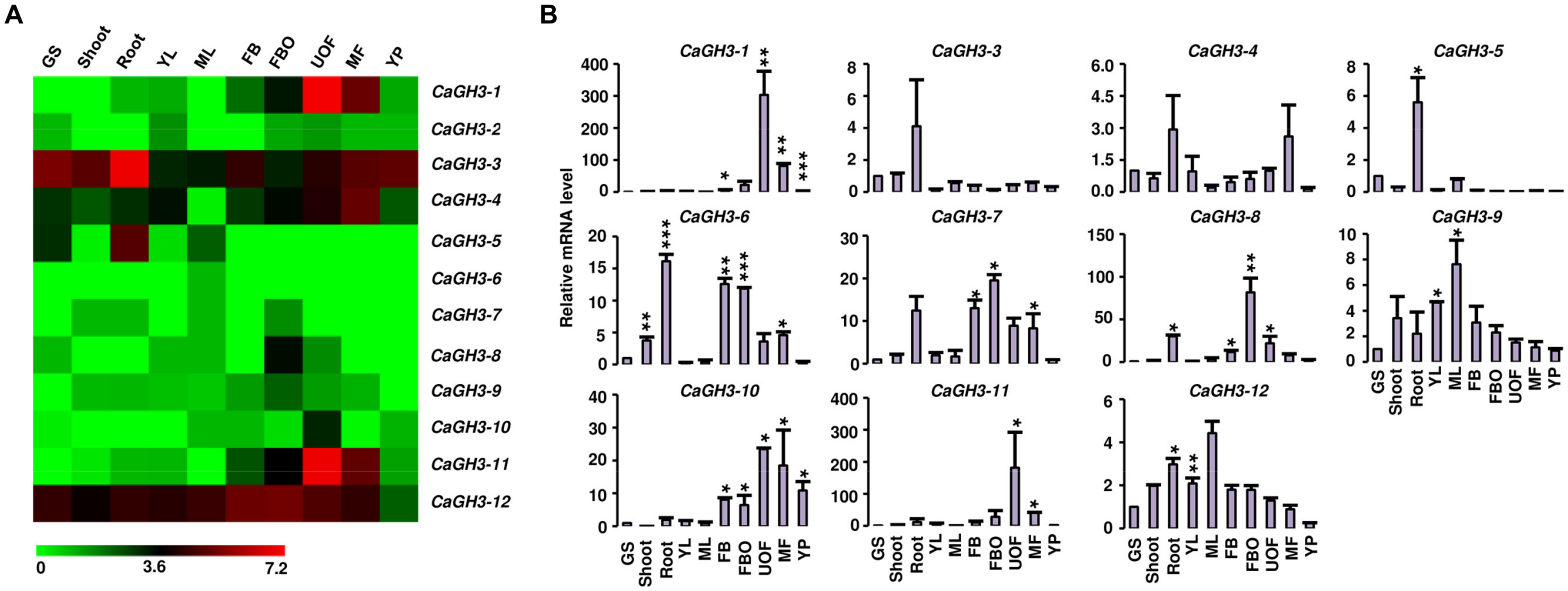
**Expression profiles of *CaGH3* genes during development. (A)** Heatmap showing expression profiles of *CaGH3* genes based on RNA-seq data in various tissues/development stages. Heatmap was generated based on log_2_ FPKM. **(B)** Real-time PCR analysis of *CaGH3* genes in various tissue/stages of development. Expression of germinating seedling (GS) was taken as a reference to determine relative mRNA level in other tissues for each gene. Error bars indicate SE of mean. *YL*, young leaf; *ML,* mature leaf; *FB,* flower bud; *UOF,* unopened flower; *FBO,* flower bud open; *MF,* mature flower; *YP,* young pod. Data points marked with asterisk (*P ≤ 0.05, **P ≤ 0.01, and ***P ≤ 0.001) indicate statistically significant difference between control (GS) and other tissues.

Phytohormone auxin is required for plant morphogenesis, including tropistic growth, root patterning, vascular tissue differentiation, axillary bud formation, and floral organ development (Zhao, 2010). Expression analysis of *GH3* genes in various tissue-types during different developmental stages in different plant species have suggested their diverse roles in plants (Gee et al., 1991; Nakazawa et al., 2001; Takase et al., 2004; Khan and Stone, 2007; Jain and Khurana, 2009; Zhang et al., 2009; Böttcher et al., 2010; Kuang et al., 2011; Kumar et al., 2012). Therefore, we performed expression analysis of *GH3* genes in various tissue/stages of development in legumes to know their putative functions. Availability of gene expression atlas covering various tissues/organs and stages of development (Benedito et al., 2008; Libault et al., 2010; Severin et al., 2010; Singh et al., 2013; Verdier et al., 2013), serves as resource to profile expression of candidate genes in legumes. We analyzed the expression of chickpea *GH3* genes using our RNA-seq data (Singh et al., 2013) and validated the results via qRT-PCR analysis (**Figure 3**). This analysis revealed that *CaGH3* genes were differentially expressed in various tissues/stages of development. *CaGH3-3* and *CaGH3-5* genes exhibited higher expression in root, which was also confirmed via qRT-PCR, suggesting their role in chickpea root development (**Figure 3**). *CaGH3-3* orthologs in *Arabidopsis*, *AtGH3-2,* and *AtGH3-6,* were found to have role in root development (Nakazawa et al., 2001; Takase et al., 2004). In addition, *CaGH3-1* and *CaGH3-11* exhibited preferential expression in unopened flower, indicating that these genes might be involved in auxin homeostasis during a specific developmental stage of flower (**Figure 3**). In rice, *OsGH3-1, -4, -5, -8,* and *-11* genes displayed highest expression level in flower (Jain et al., 2006) and *OsGH3-8* has been reported as the downstream target of rice MADS-box transcription factor (OsMADS1), which is involved in patterning of inner whorl floral organ (Prasad et al., 2005). Expression of *CaGH3-10* was also distinctly higher in unopened flower, suggesting its role in flower development. *CaGH3-10* was found to be in same phylogenetic clade with *AtGH3-5* and *-6*, whose orthologs in rice *OsGH3-1* and *-4* have higher expression in flower (Jain et al., 2006; Jain and Khurana, 2009), validating our observation. Paralogous gene pair, *CaGH3-7* and *-8* exhibited significantly higher expression in open flower bud, indicating their possible role in auxin homeostasis in early stages of flower development and support the notion that paralogs might have similar expression patterns and function. Transcript level of *CaGH3-2* could not be detected via qRT-PCR, suggesting it might be expressed in a specific tissue/stages of development. These findings highlight the role of *CaGH3* genes in overall plant development including various stages of reproductive development.

**FIGURE 4:**
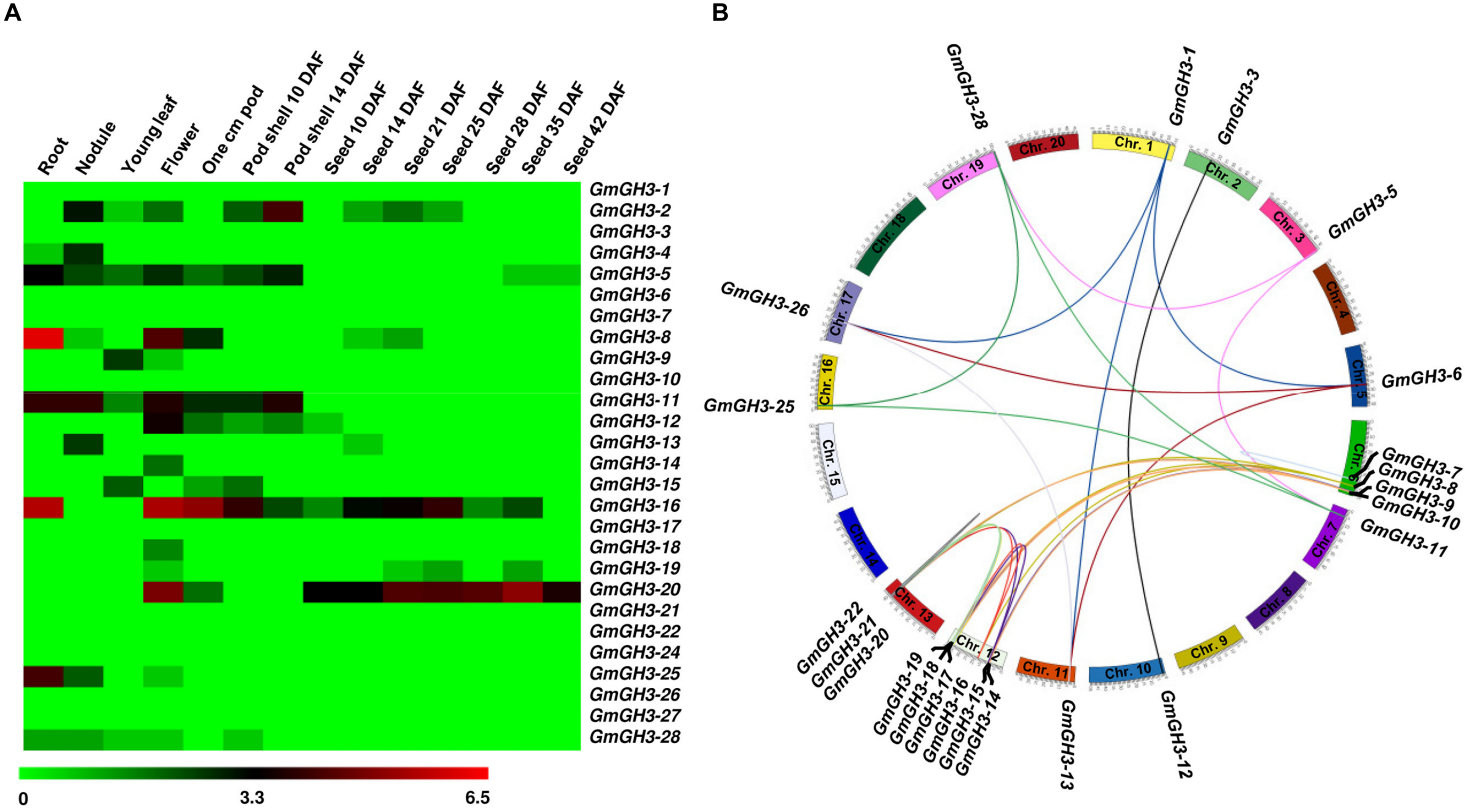
**Expression profiles and gene duplication of *GmGH3* genes. (A)** Heatmap showing expression profiles of soybean *GH3* genes at various stages of development. Heatmap was generated based on log_2_ RPKM. **(B)** Mapping of *GmGH3* genes and duplication between them are shown on the soybean chromosomes. Duplication was determined using Plant Genome Duplication Database. Genes and their duplications were mapped on chromosomes using Circos tool. Soybean chromosomes have been arranged in circle and duplications are represented by lines.

Furthermore, we analyzed the expression profiles of *GmGH3*, *MtGH3*, and *LjGH3* genes in different vegetative and reproductive tissues, utilizing expression data from published RNA-seq atlas of soybean (Severin et al., 2010), *Medicago* (Benedito et al., 2008), and *Lotus* (Verdier et al., 2013), respectively. Expression analysis of *GmGH3* genes revealed their dynamic regulation in various tissues and stages of development (**Figure 4A**). *GmGH3-8* and *GmGH3-25* showed distinctly higher expression in root, *GmGH3-4* and *GmGH3-13* were up-regulated in nodule, *GmGH3-14* and *GmGH3-18* exhibited flower-specific expression, *GmGH3-9* showed specific expression in young leaf and *GmGH3-20* expression was higher in stages of seed development (**Figure 4A**). Previously, it has been reported that *GH3* genes in soybean exhibit transient expression during floral development and higher expression in ovule and ovary at later stages of floral development (Gee et al., 1991). Reports also suggested role of *GH3* genes during seed development, for example, *GH3* gene (*YDK1*) was found to be specifically up-regulated at heart stage during embryogenesis of *Solanum chacoense* (Tebbji et al., 2010). In rice, involvement of *GH3* genes in seed development has also been reported. For instance, *OsGH3-13* overexpressing rice exhibited smaller seeds (Zhang et al., 2009) and *OsGH3-4* have higher expression during various stages of seed development (Jain and Khurana, 2009). These findings indicated that *GmGH3* genes could play an important role in seed development.

The paralogous *GmGH3* genes, *GmGH3-6* and *GmGH3-26, GmGH3-8* and *GmGH3-16, GmGH3-11* and *GmGH3-25, GmGH3-17* and *GmGH3-22*, and *GmGH3-23* and *GmGH3-24* localized on duplicated chromosomal segments, exhibited similar expression patterns in various tissues/stages of development (**Figures 4A,B**), suggesting their similar function. However, duplicated genes are also known to have a great degree of expression and functional divergence due to selection pressure and need for diversification (Prince and Pickett, 2002). Many duplicated *GmGH3* genes exhibited expression divergence as well, such as *GmGH3-1* and *GmGH3-13*, *GmGH3-3* and *GmGH3-12*, *GmGH3-5* and *GmGH3-28*, *GmGH3-9* and *GmGH3-15*, and *GmGH3-11* and *GmGH3-25* (**Figures 4A,B**). These results suggested that chromosomal duplication events not only facilitated the amplification of the *GmGH3* gene family members, but also resulted into expression divergence between duplicated genes, which might have contributed in the establishment of gene functional diversity during evolution.

Likewise, the expression of *MtGH3* and *LtGH3* genes was also found to be variable in various tissue/stages of development. For instance, *MtGH3-4* exhibited significantly higher expression in seed at 36 day after pollination (DAP), *MtGH3-8* showed greater expression in root and various stages of seed development (Supplemental Figure S2). *LjGH3-2* was found to be up-regulated in root, whereas *LjGH3-1, -6*, and *-12* showed distinctly higher expression in leaf, and *LjGH3-3, -4, -5*, and *-18* were seen to be up-regulated in root and nodule (Supplemental Figure S4). Expression of other *GH3* genes of legumes was also found to be variable in various tissue/stages of development elucidating their involvement in various growth and development processes (Wang et al., 2010; Kuang et al., 2011).

**FIGURE 5:**
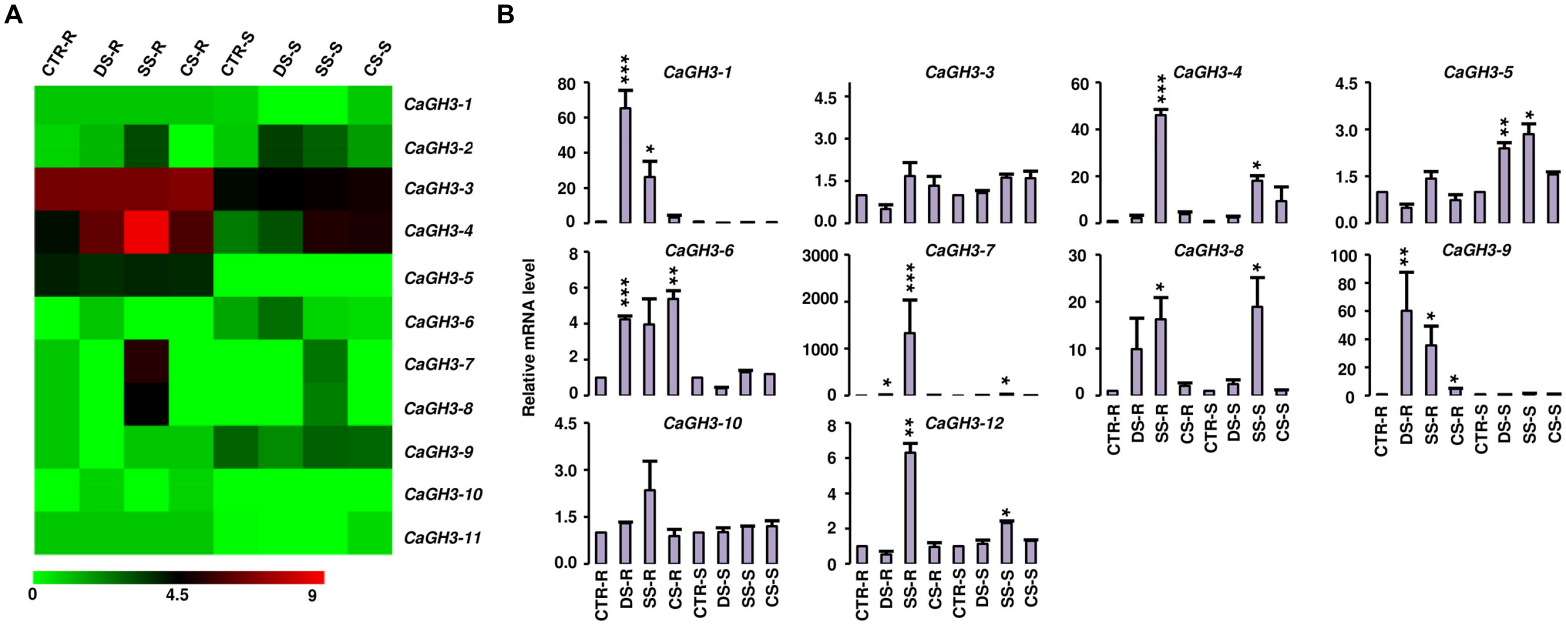
**Expression profiles of *CaGH3* genes under abiotic stress conditions. (A)** Heatmap showing expression of *CaGH3* genes based on RNA-seq data. Heatmap was generated based on log_2_ FPKM. **(B)** Real-time PCR analysis of *CaGH3* genes under various stress treatments. Root control (CTR-R) and shoot control (CTR-S) was taken as a reference to determine relative mRNA level under stress conditions. Error bars indicate standard error of mean. *DS-R*: desiccation stressed root, *SS-R,* salt stressed root; *CS-R,* cold stressed root; *DS-S,* desiccation stressed shoot; *SS-S,* salt stressed shoot; *CS-S,* cold stressed shoot. Data points marked with asterisk (*P ≤ 0.05, **P ≤ 0.01, and ***P ≤ 0.001) indicate statistically significant difference between control and stress treatments.

Furthermore, we analyzed expression patterns of paralogous/orthologous *GH3* genes to investigate their functional conservation across legumes. Although the available expression data represented diverse tissues/developmental stages in different legumes, we made an effort to define correlation in expression profiles of *GH3* genes in different legumes. Some of paralogous/orthologous *GH3* genes exhibited similar expression patterns in different legumes, such as *CaGH3-3*, *GmGH3-8*, *-16*, *-20*, *MtGH3-1* and *LjGH3-11*; *CaGH3-12*, *GmGH3-5*, *-11*, *-25*, *-28* and *LjGH3-1*; *CaGH3-5* and *GmGH3-4*; *CaGH3-4* and *LjGH3-2*; *GmGH3-13* and *LjGH3-2*; *MtGH3-2* and *LjGH3-14*; and *MtGH3-3* and *-6*, suggesting their conserved function across legumes (**Figures 3–5**; Supplemental Figures S2 and S3). Some of these paralogous/orthologous genes harbor similar cis-regulatory elements in their promoter regions (Supplemental Table S4). For instance, *CaGH3-3*, *GmGH3-8*, *-16,* and *-20* contain cis-regulatory elements, S000037, S000270, S000273, S000390, S000414, S000453, and S000461, conserved in their promoter sequences (Supplemental Table S4). An earlier study revealed similarity of gene expression profiles in various organs for a significant number of paralogous/orthologous gene pairs in *Medicago* and *Arabidopsis* (Benedito et al., 2008). Moreover, comparison of soybean transcriptome with *Medicago* and *Lotus* demonstrated similar tissue-specificity for 45% of the genes analyzed (Libault et al., 2010). Overall, these findings provide insights into the putative roles of *GH3* genes in legumes in various aspects of plant growth and development.

### DIFFERENTIAL EXPRESSION PATTERNS OF *GH3* GENES UNDER ABIOTIC STRESSES

Plants are constantly exposed to various abiotic stresses in their life cycle. Several recent studies have implicated auxin in abiotic stress responses (Jain and Khurana, 2009; Wang et al., 2010; Du et al., 2012; Kumar et al., 2012; Yuan et al., 2013). Some studies have revealed that *GH3* genes are regulated by abiotic stresses, like drought, salt, and cold stresses (Park et al., 2007; Jain and Khurana, 2009). The transcript level of *AtGH3-5* (*WES1*) has been shown to be induced by various abiotic stress conditions, like drought, high salt, and cold (Park et al., 2007). In rice, the transcript abundance of *OsGH3-1, OsGH3-2*, *OsGH3-8,* and *OsGH3-13* were markedly higher in seedlings subjected to salt, drought and cold stresses (Jain and Khurana, 2009; Zhang et al., 2009; Du et al., 2012). In *Sorghum*, at least six *GH3* genes were found to be induced upon salt and drought treatments in leaf (Wang et al., 2010).

To investigate the role of legume *GH3* genes in abiotic stress responses, we performed scanning of *cis*-acting regulatory DNA elements within promoter regions (2 kb upstream from the start codon) using PLACE database. This analysis predicted several elements responsive to auxin (IAA), abscisic acid (ABA), SA, JA, drought, salinity, and disease (Supplemental Table S4), suggesting that the function of these genes may be associated with various phytohormone signals and/or environmental stresses. Considering regulatory role of *cis*-elements, we analyzed expression of *GH3* genes under abiotic stress conditions to know their function during abiotic stresses. For chickpea, we analyzed RNA-seq data from root and shoot tissues subjected to desiccation, salinity and cold conditions (Garg et al., 2014), and performed real-time PCR analysis for validation. In our analysis, paralogous gene pair, *CaGH3-1* and *-9,* showed induction under both desiccation and salinity stresses in root (**Figures 5A,B**), and also their promoter sequences harbor desiccation (S000414) and salinity (S000453) responsive *cis*-regulatory elements (Supplemental Table S4), indicating their role in desiccation and salinity stress. Recently, rice group-I gene, *OsGH3-12*, has also been found to be markedly induced by drought stress (Du et al., 2013b). Similarly, promoter of *CaGH3-4* harbor salinity responsive *cis*-element (S000453) and showed higher expression in root under salt stress (**Figures 5A,B**). Its ortholog, *AtGH3-1*, has also been found to be up-regulated under salt stress (Sani et al., 2013), corroborating our result. Group-I paralogous genes, *CaGH3-7* and *-8*, were found to be induced in root under salinity stress (**Figures 5A,B**), implying their involvement in homeostasis of auxin under salinity stress in root. *CaGH3-5* and *-6* showed enhanced expression under desiccation, salt and cold stresses in shoot and root (**Figures 5A,B**), respectively, suggesting their role during multiple abiotic stress responses.

In *Medicago*, *MtGH3-8* and *-9* genes were induced under salt stress in root, and *MtGH3-7* was induced under drought stress in root (Supplemental Figure S2). Previous reports suggest that IAA, SA, JA, ethylene, and ABA regulate the protective responses of plants against both biotic and abiotic stress responses via signaling crosstalk (Bostock, 2005; Lorenzo and Solano, 2005; Mauch-Mani and Mauch, 2005; Ding et al., 2008; Domingo et al., 2009; Fu et al., 2011). In addition, orthologous genes, *CaGH3-10* and *MtGH3-8*, showed induced expression under salt stress in root (**Figure 5**; Supplemental Figure S2); suggesting their conserved function in both legumes. Taken together, these findings indicated that members of GH3 gene family might be involved in stress adaptation in legumes.

### HOMOLOGY MODELING AND SUBSTRATE PREFERENCES

The availability of crystal structures of two *Arabidopsis* GH3 proteins: AtGH3-12, which conjugate benzoate substrate and JA-specific AtGH3-11/JAR1 (Westfall et al., 2012); and grapevine IAA-amido synthetase GH3-1 (VvGH3-1) gave us an exciting opportunity to determine three-dimensional structure of GH3 members in legumes by homology modeling.

**FIGURE 6:**
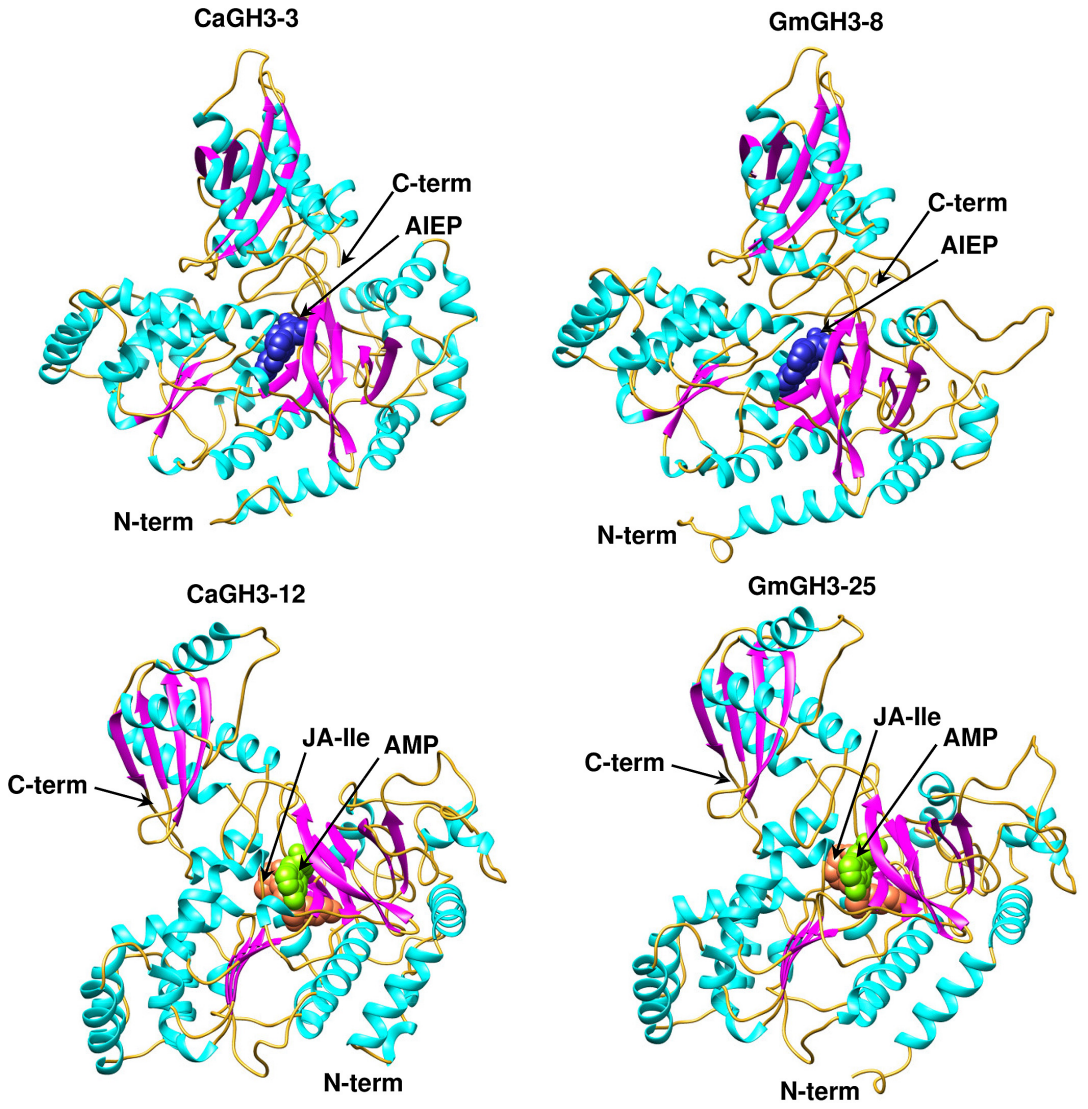
**Predicted structures of GH3 proteins.** Ribbon diagram showing the *N*- and *C*-terminal domains of chickpea (CaGH3-3 and CaGH3-8) and soybean (GmGH3-8 and GmGH3-25) GH3 protein with α-helices, β-strands and loops colored cyan, magenta, and gold, respectively. Ligands AIEP, JA-Ile, AMP are shown as space-filling model in blue, coral, and green colors, respectively.

**FIGURE 7:**
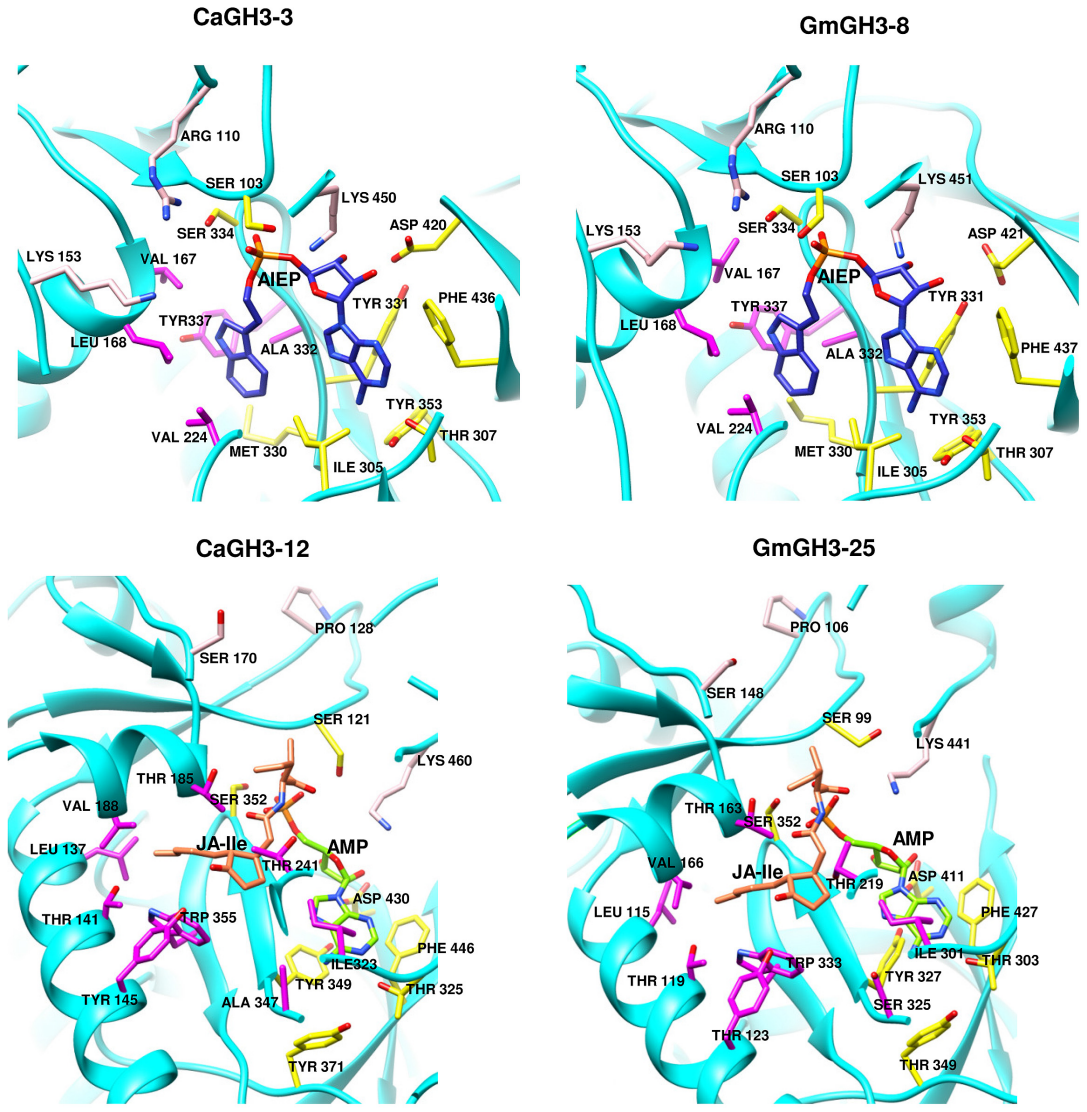
**Hormone and nucleotide binding residues in GH3 proteins.** Ribbon diagram showing hormone binding residues in magenta, nucleotide (ATP/AMP) binding residues in yellow, and residues in pink determine amino-acid preferences.

**FIGURE 8:**
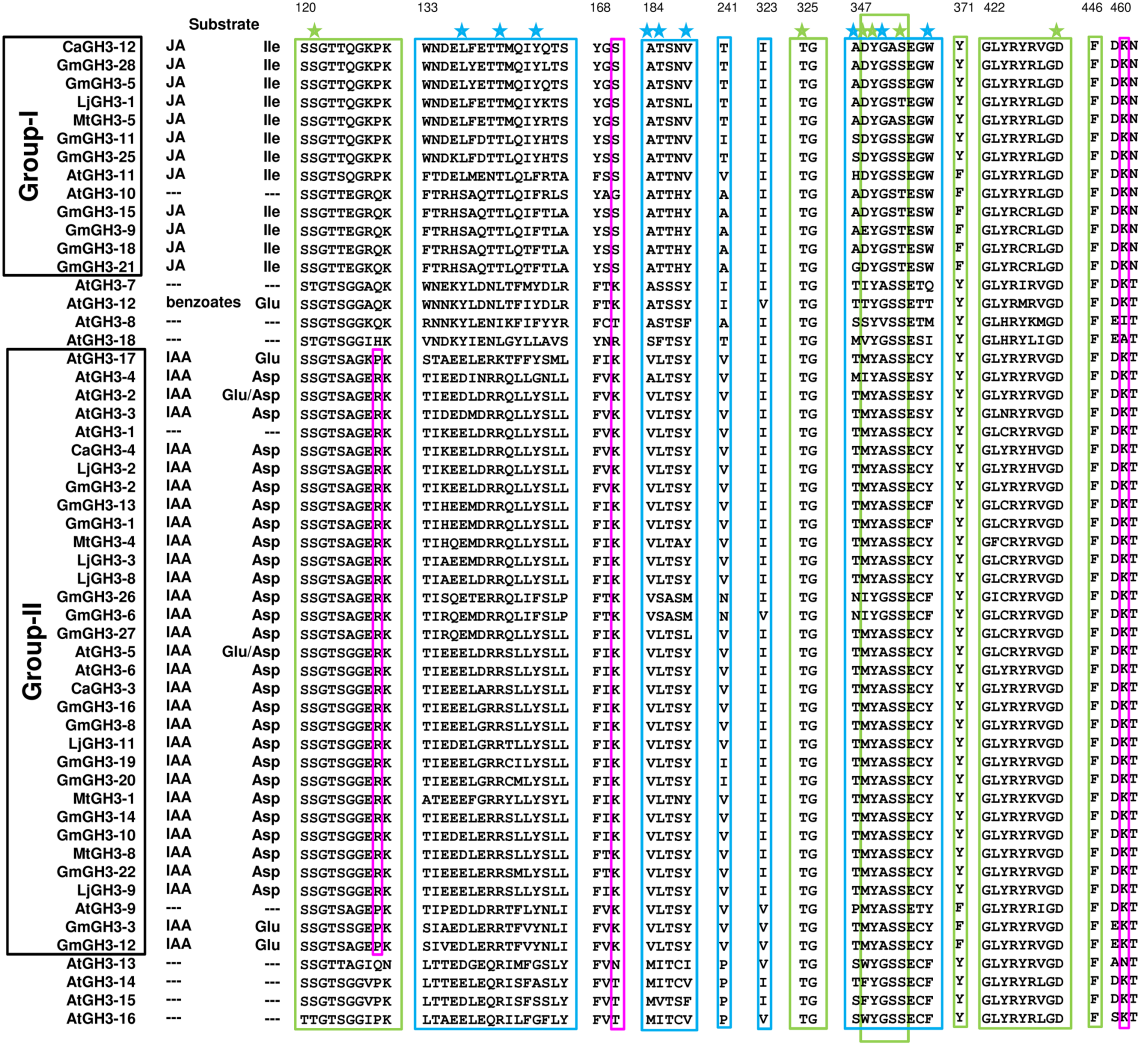
**Proposed substrates of GH3 proteins based on conserved amino acid residues.** Protein sequences of all the identified *GH3* genes were aligned using MAFFT. Green and blue boxes represent nucleotide (ATP/AMP) and hormone-binding motifs/residues, respectively. Magenta boxes represent residues determining amino-acid preferences. Only sequences with complete *C*- and *N*-terminal domains were included. Star across the top of the alignment indicates conserved residues in pocket forming active site. Numbering at the top corresponds to CaGH3-12.

Group-I protein, CaGH3-3 and GmGH3-8 of chickpea and soybean, respectively, were modeled using structure of AtGH3-11 (Protein Data Bank code 4EPL; Westfall et al., 2012) and Group-II proteins, CaGH3-12 and GmGH3-25 were modeled using grapevine, Vv-GH3-1 (Protein Data Bank code 4B2G; Peat et al., 2012). The homology modeling revealed high degree of conservation in the protein structure of these proteins. To predict active sites, we transferred ligands from template to model by superimposing structures. Ligands for group-I proteins are JA-Ile and AMP (amino acid mono phosphate), and group-II proteins are adenosine-5′-[2-(1H-indole-3-yl)ethyl]phosphate (AIEP), which mimics the adenylated intermediate of the IAA conjugation reaction (**Figures 6** and **7**; Böttcher et al., 2012; Westfall et al., 2013). By comparing sequences of model and template, we also identified the residues forming acyl acid/hormone-binding site and nucleotide binding site (**Figures 7** and **8**). Most of these residues were found to be conserved between the model and template. For example, hormone-binding residues of CaGH3-12 and GmGH3-25 with AtGH3-11 (JA-conjugating), Ca-Leu137, Gm-Leu115 to At-Leu117; Ca-Thr141, Gm-Thr119 to At-Thr121; Ca-Thr185, Gm-Thr163 to At-Thr166; Ca-Val188, Gm-Val168 to At-Val169; Ca-Ile323, Gm-Ile301 to At-Ile304; and Ca-Trp355, Gm-Trp333 to At-Trp336 are conserved (**Figures 7** and **8**). Similarly, conservation was found between hormone-binding residues of CaGH3-3 and GmGH3-8 with VvGH3-1(IAA-conjugating), Ca-Val167, Gm-Val167 to Vv-Val172; Ca-Leu168, Gm-Leu168 to Vv-Leu173; Ca-Ala332, Gm-Ala332 to Vv-Ala337; and Ca-Tyr337, Gm-Tyr337 to Vv-Tyr342 (**Figures 7** and **8**). In addition, we also found nucleotide-binding residues, Ser, Thr, Phe, and Tyr conserved in all the structures (**Figures 7** and **8**), which is in agreement with earlier studies that nucleotide binding residues conserved in not only GH3 proteins but also the ANL superfamily (Gulick, 2009; Peat et al., 2012; Westfall et al., 2012, 2013).

Further, to determine amino-acid specificities, we identified residues involved in discrimination of apolar (i.e., Ile) and acidic (i.e., Asp/Glu) substrates in the transferase reaction by comparing structures. Within the active site, a lysine residue (Lys450 in CaGH3-3, Lys460 in CaGH3-12, Lys451 in GmGH3-8 and Lys441 in GmGH3-25) was conserved at same position (**Figures 7** and **8**). This residue was also found to be highly conserved in GH3 proteins with known amino-acid preference (Westfall et al., 2012). Also, it has been found that Lys428 (AtGH3-12) is conserved in GH3-proteins that accept acidic-amino acid, whereas Ser151 (AtGH3-11) at the same position is conserved in enzymes specific to isoleucine conjugation (Westfall et al., 2012). We also found Lys153 in CaGH3-3 and GmGH3-8, suggesting CaGH3-3 and GmGH3-8 may accept acidic-amino acid (i.e., Asp/Glu) and at the same position Ser170 in CaGH3-12 and Ser148 in GmGH3-25, indicating their preferences for isoleucine (**Figures 7** and **8**). In addition, conservation of another residue Arg110 in IAA-specific CaGH3-3 and GmGH3-8 (**Figures 7** and **8**), further specified their Asp-conjugating nature. This was also corroborated by another study in grapevine, where residue Arg115 in VvGH3-1 (IAA-specific) was conserved in all the four Asp-conjugating GH3s, whereas Glu-conjugating GH3s had a Proline at that position (Peat et al., 2012). Also, the same pattern was found in IAA-conjugating GH3 enzymes with known amino-acid substrate preferences from *Arabidopsis* (Staswick et al., 2005) and rice (Zhang et al., 2009; Chen et al., 2010). Next, we also found similar conservation in residues determining amino-acid preferences for other members of GH3 proteins, which led us to propose substrates for them (**Figure 8**). Group-I proteins with conserved Ser and Lys at similar position as that of CaGH3-12 (Ser170 and Lys461; magenta boxes; **Figure 8**) are proposed to have Ile as substrate (**Figure 8**). For group-II proteins, Asp will be the substrate when Arg at 128 and Lys at positions 170 and 461 (magenta boxes; **Figure 8**); and Glu, will be the substrate when Arg is replaced by Pro at the same position (**Figure 8**). Altogether, the structures presented here showed conservation of residues at hormone-binding site, nucleotide-binding site, and amino-acid preferences determining residues, indicating similar function.

Several previous studies have reported the differential expression of *GH3* genes in various tissues/developmental stages and in response to various stimuli, including auxin, jasmonic acid, salicyclic acid, and abiotic/biotic stresses in different plants (Park et al., 2007; Zhang et al., 2007; Jain and Khurana, 2009; Kumar et al., 2012; Yuan et al., 2013; Wu et al., 2014). Our results also revealed preferential/tissue-specific and stress-responsive expression of many *GH3* genes in different legumes. The knowledge of motifs/residues of GH3 proteins that determine substrate preferences and conjugation to auxin may help modulate their binding efficiency and substrate preferences for engineering plants with desired agronomic traits.

## CONCLUSIONS

We performed a genome-wide analysis of GH3 gene family in legumes to reveal gene structure, phylogenetic relationship, and expression profiles during various developmental stages and abiotic stress conditions. Some *GH3* genes exhibited preferential/specific expression in a particular tissue and/or under abiotic stress condition(s). Our analysis revealed that *GH3* genes seem to be involved in biology of various tissues or organs and actively participate in stress responses in legumes. The analysis of protein structures of few members identified key features of substrate recognition, which might help in investigation of their molecular functions in legumes. The data generated in this study will serve as a foundation for functional characterization of GH3 gene family members in legumes.

## Conflict of Interest Statement

The authors declare that the research was conducted in the absence of any commercial or financial relationships that could be construed as a potential conflict of interest.

## Footnotes

1 http://www.phytozome.net

2 http://www.kazusa.or.jp/lotus/index.html

3 http://www.arabidopsis.org/

4 http://rice.plantbiology.msu.edu/

5 http://pfam.sanger.ac.uk/

6 http://chibba.agtec.uga.edu/duplication/

7 http://www.dna.affrc.go.jp/PLACE/

8 http://www.rcsb.org/pdb/home/home.do

9 http://www.sbg.bio.ic.ac.uk/phyre2

10 http://mordred.bioc.cam.ac.uk/∼rapper/rampage.php

11 http://soybase.org/soyseq/

12 http://mtgea.noble.org/v3/

13 http://ljgea.noble.org/v2/
