# Supplementary material for: Genome-wide analysis and expression profiling suggest diverse roles of GH3 genes during development and abiotic stress responses in legumes
Source: Front Plant Sci. 2015 Jan 14;5:789. doi: 10.3389/fpls.2014.00789 (PMC4294127; doi:10.3389/fpls.2014.00789)
Supplement: Supplementary file 1 [file Presentation_1.PDF]

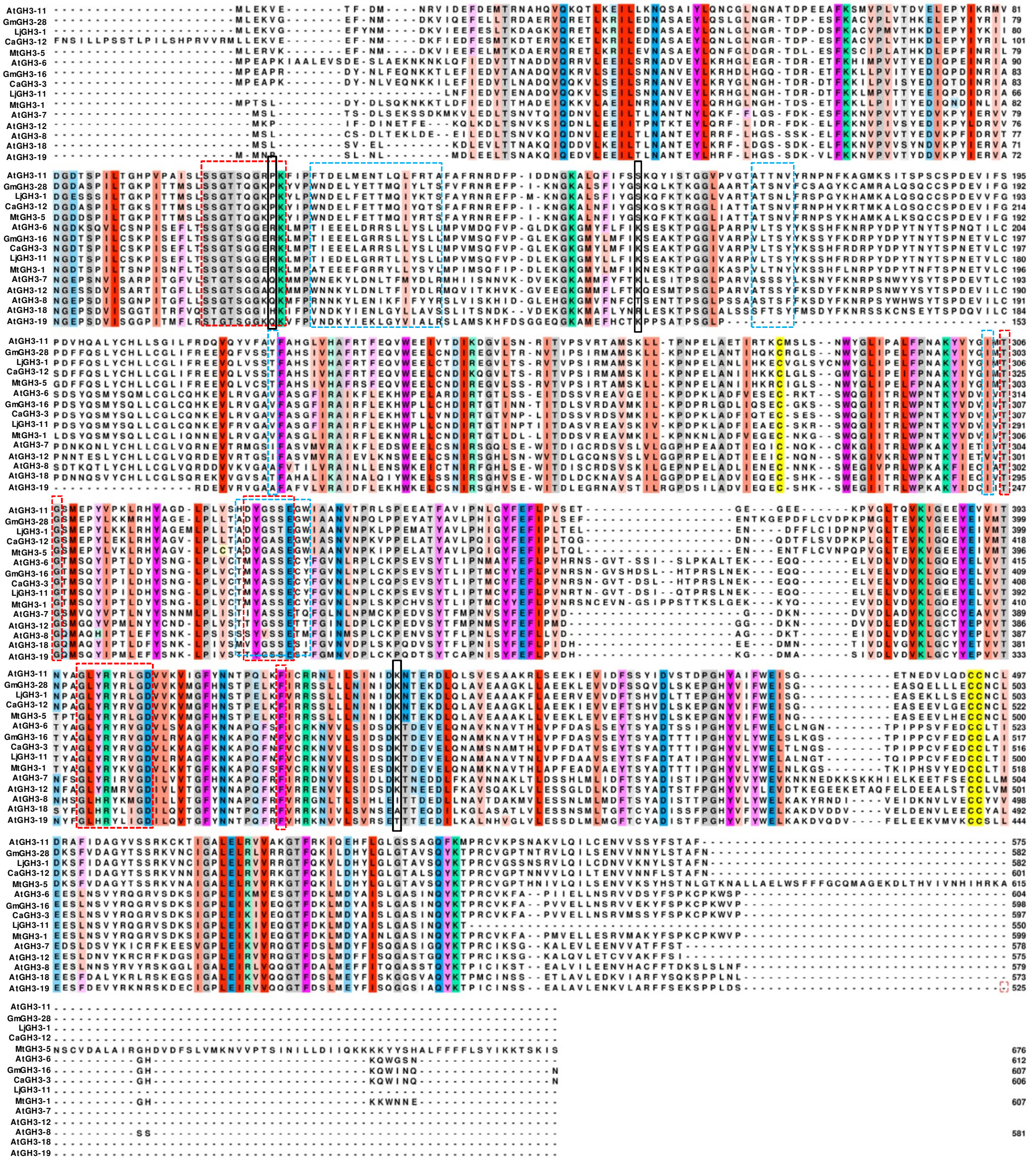

Supplemental Figure S1 Multiple sequence alignment of full-length protein sequences of CaGH3-3, and -12, GmGH3-16, and -28, MtGH3-1, and -5, LjGH3-1, and -11, and AtGH3-6, -7, -8, -11, -12, -18 and -19 obtained with MAFFT. Residues conserved in more than 50% of aligned sequences are highlighted with colors. Gaps (*dashes*) have been allowed to maximize the alignments. Red and blue boxes represent nucleotide (ATP/AMP) and hormone-binding motifs/residues, respectively. Black boxes represent residues governing amino-acid preferences.

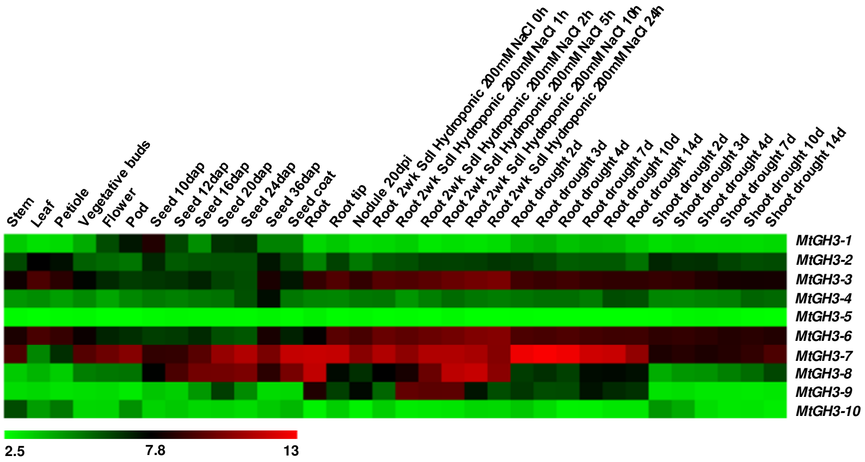

Supplemental Figure S2 Heatmap showing expression profiles of *Medicago GH3* genes at various stages of development and stress treatments. Expression data was taken from MtGEA and transformed on $\log_2$ scale for generating heatmap using MeV.

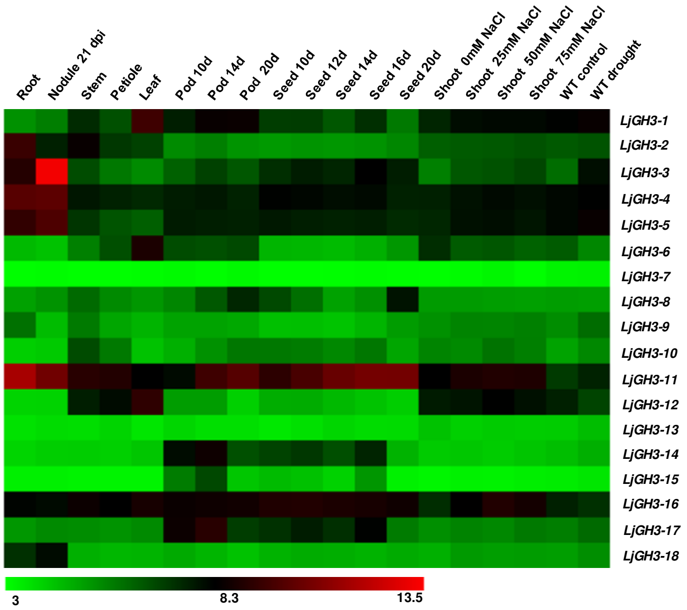

Supplemental Figure S3 Heatmap showing expression profiles of *Lotus GH3* genes at various stages of development and stress treatments. Expression data was taken from LjGEA and transformed on $\log_2$ scale for generating heatmap using MeV.
.

Supplemental Table S1 Primer sequences of chickpea GH3 genes used for qRT-PCR.

Gene name	Primer sequence
CaGH3-1	F-AAACTCCTAGGTGCACCAACAAC<br>R-ACGTTTTAATGGTGCATGCATT
CaGH3-2	F-AGCCGTTCTATGTCTCGATTCATA<br>R-GCACGACAACAGTTCCATTTGA
CaGH3-3	F-GAGTCTCTCAACAGCGTTTATCGA<br>R-CCACAATCTTTATCTCAAGTGGACCTA
CaGH3-4	F-CGCCAACTTCTTTACAGCCTTCT<br>R-CTTGTCCAATCCCGGAACAT
CaGH3-5	F-AAGAACGCCAAACATCACCAA<br>R-GCGAACAGAGGCATTATGAAGA
CaGH3-6	F-AAGCCCTGACCAAACCATTCT<br>R-GCTAGGAGTTGACAATGCATGATT
CaGH3-7	F-CAAGGGATTATGACAGGTGTTATGG<br>R-CAAAGGAACATCTCCAGCATAATG
CaGH3-8	F-ACCGACCGACGAACTTCTTG<br>R-GTGTAGCCAGGATCAACAAAAGACT
CaGH3-9	F-CAAAGGCAGAGGTTGTTGATTTT<br>R-AATAACATAATGTCCCGGTTGGTT
CaGH3-10	F-CAACGCCCTTGTTACTGAAAATT<br>R-CAGCAAGATTTTTGTTAGGTTTAAGGA
CaGH3-11	F-GGTTCAAATTGGACAAGAGTACGA<br>R-TCATAGAAGAGAAGAAGAATGAGAATTTACC
CaGH3-12	F-CAAGAGGTGGTCTGATAGCAACA<br>R-TGTGCGTTTGTAATGTGGATTG

Supplemental Table S2 List of *GH3* genes identified in chickpea, soybean, *Medicago* and *Lotus* and their sequence characteristics.

Gene name	Gene identifier	Chr./Scaffolds	Start	Stop	Strand	Gene length (bp)	ORF length (bp)	Number of exons	Protein Length (aa)	Protein Mol. Wt. (kDa)	Protein pI
CaGH3-1	Ca_00851.1	1	11127371	11131251	+	3881	972	3	323	36.10	6.94
CaGH3-2	Ca_03311.1	3	16901364	16899454	-	1911	330	3	109	12.43	5.71
CaGH3-3	Ca_09034.1	scaffold06944	216164	219344	+	3181	1821	3	606	68.49	5.96
CaGH3-4	Ca_09540.1	scaffold01924	49262	46791	-	2472	1806	3	601	68.46	6.17
CaGH3-5	Ca_10695.1	scaffold00077	133194	130993	-	2202	969	5	322	36.87	6.42
CaGH3-6	Ca_16439.1	scaffold01790	43288	44045	+	758	690	2	229	26.08	7.89
CaGH3-7	Ca_16627.1	scaffold01899	18458	14863	-	3596	1419	4	472	53.20	6.04
CaGH3-8	Ca_16628.1	scaffold01899	48408	46356	-	2053	1206	2	401	45.26	5.39
CaGH3-9	Ca_17370.1	scaffold02395	42966	44512	+	1547	582	2	193	22.11	9.07
CaGH3-10	Ca_18349.1	scaffold03415	17044	14961	-	2084	1500	2	499	56.84	5.86
CaGH3-11	Ca_20618.1	scaffold15418	2026	565	-	1462	999	1	332	38.42	8.32
CaGH3-12	TC15657	from transcriptome	--	--	--	--	1806	--	601	67.59	6.09
GmGH3-1	Glyma01g39780.1	1	51645379	51647678	+	2298	1740	3	579	65.85	5.70
GmGH3-2	Glyma02g13910.1	2	12247014	12249762	+	2747	1788	3	595	67.5	5.89
GmGH3-3	Glyma02g17360.1	2	15651184	15647015	-	4170	1821	4	606	69.03	6.88
GmGH3-4	Glyma03g30590.1	3	38500737	38497213	-	3525	1731	3	576	65.03	5.64
GmGH3-5	Glyma03g41700.1	3	47100629	47104968	+	4338	1716	4	571	64.41	6.08
GmGH3-6	Glyma05g21680.1	5	26473856	26476824	+	2967	1785	3	594	67.08	5.44
GmGH3-7	Glyma06g37401.1	6	39819569	39821049	+	1479	354	3	117	13.52	5.40
GmGH3-8	Glyma06g40860.1	6	44086613	44089781	+	3167	1830	3	609	68.87	6.41
GmGH3-9	Glyma06g45120.2	6	47927128	47921881	-	5248	1749	4	582	65.6	6.55
GmGH3-10	Glyma06g45640.1	6	48387184	48391475	+	4290	1875	3	624	70.24	5.71
GmGH3-11	Glyma07g06370.1	7	5099080	5094245	-	4836	1749	4	582	65.47	5.68
GmGH3-12	Glyma10g02440.1	10	1707855	1712188	+	4332	1827	4	608	69.15	6.52
GmGH3-13	Glyma11g05510.1	11	3847544	3849971	-	2426	1782	3	593	67.35	5.71
GmGH3-14	Glyma12g11200.1	12	9219566	9215570	-	3997	1890	3	629	70.97	6.02
GmGH3-15	Glyma12g11890.1	12	10025466	10030865	+	5398	1842	4	613	68.96	6.38
GmGH3-16	Glyma12g17510.1	12	17535434	17532260	-	3175	1824	3	607	68.75	6.26
GmGH3-17	Glyma12g32410.1	12	35904564	35900392	-	4173	1857	3	618	68.99	5.58
GmGH3-18	Glyma12g32910.1	12	36306326	36311860	+	5533	1962	4	653	72.99	6.02
GmGH3-19	Glyma12g34480.2	12	37631029	37627772	-	3258	1638	5	545	61.68	5.51
GmGH3-20	Glyma13g36030.1	13	37394685	37398432	+	3746	1836	4	611	69.08	6.31
GmGH3-21	Glyma13g37550.1	13	38607815	38602953	-	4863	1800	4	599	67.05	5.81
GmGH3-22	Glyma13g38000.1	13	38936763	38941178	+	4414	1893	3	630	70.76	5.23
GmGH3-23	Glyma15g23205.1	15	22250431	22251055	+	625	294	3	97	11.00	10.4
GmGH3-24	Glyma15g38731.1	15	45192416	45193109	+	692	303	1	100	11.48	5.03
GmGH3-25	Glyma16g03011.1	16	2587998	2583259	-	4740	1749	3	582	65.13	5.99
GmGH3-26	Glyma17g18040.1	17	15221030	15218293	-	2738	1782	3	593	66.87	5.30
GmGH3-27	Glyma17g18080.1	17	15288847	15286734	-	2114	1476	3	491	55.45	5.59
GmGH3-28	Glyma19g44310.1	19	49775282	49780336	+	5053	1749	4	582	65.65	5.83

Supplemental Table S2 Cont.

Gene name	Gene identifier	Chr./Scaffolds	Start	Stop	Strand	Gene length (bp)	ORF length (bp)	Number of exons	Protein		
									Length (aa)	Mol. Wt. (kDa)	pI
MtGH3-1	Medtr2g081860.1	2	24684511	24687603	+	3091	1824	3	607	68.75	6.08
MtGH3-2	Medtr3g023280.1	3	6658152	6657698	-	455	387	2	128	14.66	8.88
MtGH3-3	Medtr5g016310.1	5	5565017	5568425	+	3407	2457	5	818	94.34	9.68
MtGH3-4	Medtr5g016320.1	5	5576809	5574506	-	2304	1803	3	600	68.17	5.46
MtGH3-5	Medtr7g117110.1	7	38127792	38133586	+	5793	2031	7	676	76.42	7.24
MtGH3-6	Medtr8g037720.1	8	8749009	8747928	-	1082	897	3	298	33.30	7.00
MtGH3-7	Medtr8g037730.1	8	8749741	8749200	-	542	387	2	128	14.59	5.55
MtGH3-8	AC229702_19.1	scaffold AC229702	66878	64951	-	1928	1521	2	506	57.11	5.53
MtGH3-9	AC229702_20.1	scaffold AC229702	69716	69211	-	506	420	2	139	15.81	6.61
MtGH3-10	AC233100_38.1	scaffold AC233100	139561	141732	+	2170	1188	7	395	--	--
LjGH3-1	chr1.CM0105.550.r2.d	1	65720787	65723693	+	2905	1749	4	582	65.68	6.03
LjGH3-2	chr2.CM0124.1040.r2.m	2	19453046	19450951	-	2096	1806	3	601	68.08	5.53
LjGH3-3	chr2.CM0250.160.r2.m	2	28758418	28760436	+	2017	1812	3	603	68.46	5.25
LjGH3-4	chr3.CM0406.430.r2.d	3	32988949	32990111	+	1161	723	3	240	27.19	5.57
LjGH3-5	chr3.CM0406.470.r2.d	3	33024864	33022996	-	1869	831	2	276	30.81	5.27
LjGH3-6	chr3.CM0711.400.r2.d	3	37195234	37190831	-	4404	1788	4	595	67.28	8.85
LjGH3-7	chr4.CM0042.2280.r2.m	4	43015883	43015346	-	538	528	1	175	19.54	9.48
LjGH3-8	chr4.LjT45J24.60.r2.m	4	12994066	12992088	-	1979	1794	3	597	67.51	5.75
LjGH3-9	LjSGA_016734.2	scaffold	1	2820	+	2818	1719	3	572	64.33	5.95
LjGH3-10	LjSGA_017327.1	scaffold	775	2560	+	1784	1506	2	501	56.34	6.9
LjGH3-11	LjSGA_019565.1	scaffold	2526	484	-	2043	1653	3	550	61.84	5.13
LjGH3-12	LjSGA_025175.1	scaffold	1198	1	-	1198	558	3	185	20.64	6.29
LjGH3-13	LjSGA_029406.3	scaffold	1290	1844	+	553	474	2	157	18.08	5.28
LjGH3-14	LjSGA_040454.1	scaffold	1640	1212	-	429	429	1	142	16.09	5.15
LjGH3-15	LjSGA_048646.1	scaffold	314	1	-	314	312	1	103	11.88	6.29
LjGH3-16	LjSGA_075780.1	scaffold	1197	704	-	494	492	1	163	18.26	5.49
LjGH3-17	LjSGA_096861.1	scaffold	758	1	-	758	756	1	251	28.23	7.97
LjGH3-18	LjSGA_142965.1	scaffold	783	1	-	783	777	1	258	28.87	6.52

Supplemental Table S3 Percentage identity among full-length protein sequences of (a) CaGH3 and (b) GmGH3.

(a)

	CaGH3-1	CaGH3-2	CaGH3-3	CaGH3-4	CaGH3-5	CaGH3-6	CaGH3-7	CaGH3-8	CaGH3-9	CaGH3-10	CaGH3-11	CaGH3-12
CaGH3-1	***	1.6	13.8	13.9	4.9	4.5	23.2	26.9	44.6	16.6	6.8	20.1
CaGH3-2		***	11.4	12.5	3.0	11.6	3.2	1.9	0.4	13.2	4.0	3.5
CaGH3-3			***	62.9	12.1	19.8	26.3	25.1	11.5	48.6	14.5	32.5
CaGH3-4				***	12.1	19.3	27.7	26.8	11.5	48.6	14.5	34.3
CaGH3-5					***	11.3	10.0	9.7	2.5	8.4	12.0	12.5
CaGH3-6						***	13.9	14.4	1.3	23.2	19.3	11.1
CaGH3-7							***	76.9	18.6	28.1	26.2	52.4
CaGH3-8								***	23.1	29.0	24.6	47.7
CaGH3-9									***	13.2	1.5	16.3
CaGH3-10										***	17.9	26.3
CaGH3-11											***	22.5
CaGH3-12												***

(b)

	GmGH3-1	GmGH3-2	GmGH3-3	GmGH3-4	GmGH3-5	GmGH3-6	GmGH3-7	GmGH3-8	GmGH3-9	GmGH3-10	GmGH3-11	GmGH3-12	GmGH3-13	GmGH3-14	GmGH3-15	GmGH3-16	GmGH3-17	GmGH3-18	GmGH3-19	GmGH3-20	GmGH3-21	GmGH3-22	GmGH3-23	GmGH3-24	GmGH3-25	GmGH3-26	GmGH3-27	GmGH3-28
GmGH3-1	***	76.2	50.5	29.7	36.6	78.6	7.8	62.0	29.5	58.4	39.2	51.0	94.9	58.2	30.9	62.7	55.3	28.1	54.8	61.0	30.2	58.7	3.1	6.1	38.7	78.6	66.2	36.7
GmGH3-2		***	51.1	28.7	35.3	72.5	6.9	63.2	30.4	59.7	36.3	51.5	77.3	59.2	32.0	63.5	56.7	29.4	55.9	61.9	30.8	59.3	2.8	5.5	35.8	73.0	61.2	35.2
GmGH3-3			***	29.5	34.1	48.3	7.3	50.2	31.0	49.1	34.6	95.7	50.1	49.2	32.7	50.2	46.2	29.1	45.9	51.0	31.4	48.8	2.5	5.9	34.6	48.0	41.3	34.1
GmGH3-4				***	31.6	29.0	6.6	27.3	26.9	28.5	31.6	29.2	29.0	28.6	28.7	27.6	25.5	27.8	25.6	27.8	29.8	26.9	2.8	5.0	31.4	29.6	26.0	31.4
GmGH3-5					***	36.0	19.4	33.9	45.1	34.2	80.2	34.0	36.0	33.7	48.0	33.4	33.1	44.0	30.1	33.0	46.4	34.3	3.8	16.5	79.4	36.2	31.1	95.4
GmGH3-6						***	7.1	58.1	30.4	56.3	36.6	48.6	78.7	56.0	32.2	58.4	52.8	28.3	51.7	57.7	31.0	55.6	3.2	5.8	36.8	90.4	71.0	33.8
GmGH3-7							***	7.2	8.4	7.4	16.7	7.2	7.2	7.5	8.0	6.9	7.6	7.7	8.0	7.4	8.5	7.3	16.9	51.1	16.2	7.4	8.3	18.6
GmGH3-8								***	30.1	72.1	35.4	50.8	62.1	72.1	32.0	97.4	68.7	29.0	74.8	85.2	31.4	70.8	3.1	6.2	35.9	58.5	50.6	33.9
GmGH3-9									***	31.0	45.3	31.2	29.5	30.5	88.1	30.1	28.7	69.4	27.0	29.7	73.4	30.6	1.7	6.7	45.9	30.7	25.6	44.5
GmGH3-10										***	34.2	49.1	58.7	92.1	32.6	72.5	77.3	30.0	61.8	69.7	31.8	78.9	3.4	6.3	34.6	56.3	50.4	33.9
GmGH3-11											***	34.5	38.5	34.2	48.2	35.0	34.3	43.8	32.4	35.2	46.5	36.1	3.3	13.2	94.9	36.2	31.5	81.4
GmGH3-12												***	50.6	49.3	32.6	50.8	46.2	29.0	46.2	51.5	31.3	48.9	2.8	5.8	34.5	48.4	41.0	34.0
GmGH3-13													***	58.5	30.9	62.5	55.5	28.4	55.2	61.6	29.9	58.6	3.2	6.5	37.7	78.7	66.0	36.1
GmGH3-14														***	32.0	71.9	76.4	29.7	61.1	69.1	31.3	78.1	3.5	63.4	34.3	56.2	50.3	33.4
GmGH3-15															***	32.1	30.8	74.5	29.1	31.4	77.4	32.3	1.8	6.4	48.5	32.2	27.2	47.7
GmGH3-16																***	68.6	29.1	75.7	86.1	31.6	70.4	2.9	5.9	35.7	58.7	51.1	33.4
GmGH3-17																	***	28.3	57.1	65.2	30.0	91.4	3.2	6.3	34.4	52.8	46.3	32.9
GmGH3-18																		***	27.1	28.9	84.7	28.9	1.4	6.3	44.0	28.7	24.7	43.8
GmGH3-19																			***	85.5	29.2	60.0	2.5	6.7	32.2	51.9	52.1	30.1
GmGH3-20																				***	30.9	67.8	2.8	6.1	35.2	58.1	49.8	33.0
GmGH3-21																					***	30.6	1.3	6.5	47.2	31.0	26.0	46.2
GmGH3-22																						***	3.2	6.0	36.2	55.6	48.4	34.0
GmGH3-23																							***	11.9	3.4	3.0	3.8	4.1
GmGH3-24																								***	13.1	6.2	6.8	15.8
GmGH3-25																									***	36.4	31.7	80.4
GmGH3-26																										***	71.0	36.0
GmGH3-27																											***	30.7
GmGH3-28																												***

Supplemental Table S4 *Cis*-regulatory elements identified in the promoter (2 kb upstream sequence from the start codon) sequence of Ca*GH3* and *GmGH3* genes.

S. No.	Motif sequence	PLACE motif identity	Motif name	*CaGH3* genes	*GmGH3* genes
1	TGACG	S000024	TGA-box (Auxin and SA responsive)	2, 4, 7, 9	1, 3, 4, 6, 7, 10, 12, 13, 16, 17, 18, 22, 23, 25, 26, 27, 28
2	TGTCTC	S000270	ARFAT (ARF binding domain)	1, 2, 3, 4, 5, 7, 8, 10	1, 3, 4, 5, 6, 8, 9, 13, 16, 17, 18, 19, 20, 21, 24, 25, 26, 27
3	TGACGTAA	S000234	AUXRE (Auxin responsive)		6, 26
4	TGACGTGGC	S000235	AUXRE (Auxin responsive)		6, 26, 27
5	AWTTCAAA	S000037	ERELEE4 (Ethylene responsive)	3, 4, 6, 8, 10, 11	2, 3, 4, 6, 7, 8, 9, 10, 11, 12, 14, 15, 16, 18, 19, 20, 21, 24, 27, 28
6	TTGAC	S000390	WBOXATNPR1 (SA-responsive)	1, 2, 3, 4, 5, 6, 7, 8, 9, 10	1, 2, 3, 4, 5, 6, 7, 8, 9, 10, 11, 12, 13, 14, 15, 16, 17, 18, 19, 20, 21, 22, 23, 24, 25, 26, 27, 28
7	CACGTG	S000042	ABA-responsive	1, 6, 9	2, 5, 7, 15, 16, 26, 27
8	GAAAAA	S000453	GT1GMSCAM4 (Salt-responsive)	1, 2, 3, 4, 5, 6, 7, 8, 9, 10, 11	1, 2, 3, 4, 5, 6, 7, 8, 9, 10, 11, 12, 13, 14, 15, 16, 17, 18, 19, 20, 21, 22, 23, 24, 25, 26, 27, 28
10	CCGAC	S000153	LTRECOREATCOR15 (Cold/Drought-responsive)	1, 2, 8	3, 5, 6, 7, 9, 11, 12, 13, 17, 22, 23, 24, 27, 28
11	ACTTTA	S000273	NTBBF1ARROLB (tissue-specific expression and auxin-responsive)	1, 3, 4, 5, 6, 7, 8, 9, 10, 11	2, 3, 4, 7, 9, 10, 12, 13, 14, 15, 16, 17, 18, 19, 20, 21, 22, 23, 24, 25, 27, 28
12	CATATG	S000370	auxin-responsive	2, 4, 5, 9	2, 4, 15, 17, 18, 19, 23, 27
13	AACGTG	S000458	T/GBOXATPIN2 (JA-responsive)	1, 2, 4, 9	2, 4, 7, 8, 9, 10, 11, 13, 16, 20, 21, 23, 24
14	ACGTG	S000414	ABRELATERD1 (Drought-responsive)	1, 2, 3, 4, 6, 7, 9, 10	2, 3, 5, 6, 7, 8, 9, 10, 11, 12, , 13, 14, 15, 16, 18, 20, 21, 22, 23, 24, 26, 27, 28
15	CACATG	S000174	Drought-responsive	2, 3, 4, 6, 7, 8, 9	3, 4, 6, 10, 12, 15, 16, 17, 18, 22, 23, 26, 27, 28
17	AAAGAT	S000461	Disease-responsive	2, 3, 4, 6, 7, 8, 9, 10	2, 3, 4, 5, 6, 7, 8, 9, 10, 11, 13, 14, 15, 16, 17, 18, 19, 20, 21, 22, 23, 24, 25, 26, 27
18	TTGACC	S000142	Disease-responsive	2, 3, 7, 9	2, 3, 5, 9, 19, 24, 25
